# The Story of the Insane from Year to Year

**Published:** 1902-11-01

**Authors:** 


### THE STORY OF THE INSANE FROM YEAR TO YEAR.

Fifteenth Annual Series.

### JOINT COUNTIES' ASYLUM, CARMARTHEN.

Here the number of patients on January 1st was 633, and on December 31st 648. There were 99 first admissions, 10 readmissions, 35 discharged recovered, 7 discharged relieved, and 50 deaths. The average number resident during the year was 637, and the total number under treatment was 742. Calculated on the admissions the recoveries reach the creditable percentage of 42·21, and calculated on the average number resident the deaths are 7·81 per cent. Of the year's deaths 9 were caused by cerebral and spinal diseases, 18 by consumption, and 4 by other lung diseases. Of the admissions various moral causes are supposed to account for the insanity in 10 instances; and among the physical causes hereditary influence comes first with 37 cases, and intemperance in drink next with 15. The asylum now contains 6 more patients than it is intended for; but there are 69 in residence chargeable to other counties, and should the pressure on space become too great some of these could be transferred to their own or other asylums where room could be found. Dr. Goodall remarks that the cases admitted were not very satisfactory from the point of view of treatment, and like so many other medical superintendents he points out the fact that "patients still require to be brought for treatment much sooner than they are." Forty per cent. of the year's deaths were caused by tuberculous disease, and it is not to be wondered at that Dr. Goodall recommends the segregation of consumptive patients, although he recognises the difficulties in the way of a solution of the problem. In an asylum of 600 beds the difficulties are considerable, not so much in the provision of suitable accommodation for nothing is better for the treatment of phthisis than cheap wooden buildings, but in the extra expense involved by an increased nursing staff. When referring to private patients, Dr. Goodall states that a much larger number would probably be sent in, if suitable separate accommodation could be provided for them. This is unquestionable, and when one thinks of the profits which the joint counties would derive from a block containing 70 or 80 patients, it is a marvel that land is not procured, and the building erected. Dr. Goodall has two assistant medical officers, one of these, we are pleased to note, being a lady. The committee "bear testimony to the efficient and able management of Dr. Goodall." We should much like to learn that the condition relative to the non-giving of pensions had been abolished. The weekly rate for in-county patients is nearly 8s. 8d. per head. Out-county cases are charged 13s. to 14s., and private patients from 10s. to 40s.

### WORCESTER COUNTY ASYLUM.

This asylum contained 1,169 patients on January 1st, and 1,180 on the last day of December. There were 210 first admissions, 36 readmissions, 97 recoveries, 13 discharged relieved, and 108 died. The average number resident was 1,174, and the total number under treatment was 1,402. The recoveries stand at 43·3 per cent. of the admissions, and the deaths at 9·1 per cent. of the average number resident. The former percentage is high, and the latter is about the average in kindred institutions. Twenty-nine of the deaths were due to cerebral and spinal diseases, 10 to consumption, 20 to pneumonia, and 13 to colitis. Of the 246 admissions it is stated that 15 owed their insanity to moral causes, 57 to hereditary influence or family predisposition, 47 to previous attacks, and 19 to intemperance in drink. Dr. Braine-Hartnell and his assistants have had an unusual amount of surgical practice during the year for there were 12 cases of fractured bones. One of these was caused in a struggle with a criminal patient, and it is a curious fact that these criminal patients are hopelessly incompatible with the average run of cases in our county asylums. There was one case of typhoid fever, the origin of which could not be traced. We sympathise with the medical staff in the trouble they have had with that disease—colitis. It has an odd way of appearing and disappearing, and it seems often quite impossible to account for either the one or the other. Dr. Hartnell says, "The idea put forward by the Commissioners in Lunacy, that infection is distributed by means of vegetables which have been watered with liquid sewage, does not appear to me to be tenable." We also think that the Commissioners' idea is not a very probable one. Such an easily found cause, if it be a cause, would long since have been detected; besides, in some asylums every article of diet has been omitted in turn without result. Dr. Hartnell is very guarded in his views as to the employment of nurses in the male wards, and rightly thinks that "we ought to be able to show that the increased cost would give an increased recovery rate before we make such wide and sweeping changes"; and increase of cost is not the only objection that will occur to the asylum physician. A third assistant medical officer was appointed during the year, and we think the committee and the medical superintendent made a mistake in not appointing a lady. Where three assistants are in residence there need never be any serious difficulty in the way, and the advantages are so great that they far outweigh any little drawbacks. The committee "desire to testify their appreciation of the manner in which these gentlemen [the medical staff] have carried out the responsible duties of their respective offices." The weekly cost of maintenance was 8s. 2d. per head.

